# RTP801/REDD1 Is Involved in Neuroinflammation and Modulates Cognitive Dysfunction in Huntington’s Disease

**DOI:** 10.3390/biom12010034

**Published:** 2021-12-27

**Authors:** Leticia Pérez-Sisqués, Júlia Solana-Balaguer, Genís Campoy-Campos, Núria Martín-Flores, Anna Sancho-Balsells, Marcel Vives-Isern, Ferran Soler-Palazón, Marta Garcia-Forn, Mercè Masana, Jordi Alberch, Esther Pérez-Navarro, Albert Giralt, Cristina Malagelada

**Affiliations:** 1 Departament de Biomedicina, Facultat de Medicina i Ciències de la Salut, Institut de Neurociències, Universitat de Barcelona, 08036 Barcelona, Spain; leticiaperezsisques@gmail.com (L.P.-S.); julia.solbal@gmail.com (J.S.-B.); geniscampoy@hotmail.com (G.C.-C.); nuria.martinfl21@gmail.com (N.M.-F.); asanchobalsells@gmail.com (A.S.-B.); mvives1304@gmail.com (M.V.-I.); ferransoler@outlook.com (F.S.-P.); marta.garcia-forn@mssm.edu (M.G.-F.); mmasana@ub.edu (M.M.); alberch@ub.edu (J.A.); estherperez@ub.edu (E.P.-N.); 2 Institut d’Investigacions Biomèdiques August Pi i Sunyer (IDIBAPS), 08036 Barcelona, Spain; 3 Centro de Investigación Biomédica en Red Sobre Enfermedades Neurodegenerativas (CIBERNED), 28031 Madrid, Spain; 4 Production and Validation Center of Advanced Therapies (Creatio), Faculty of Medicine and Health Science, Universitat de Barcelona, 08036 Barcelona, Spain

**Keywords:** RTP801/REDD1, Huntington’s disease, neuroinflammation, hippocampus, cognitive dysfunction

## Abstract

RTP801/REDD1 is a stress-regulated protein whose levels are increased in several neurodegenerative diseases such as Parkinson’s, Alzheimer’s, and Huntington’s diseases (HD). RTP801 downregulation ameliorates behavioral abnormalities in several mouse models of these disorders. In HD, RTP801 mediates mutant huntingtin (mhtt) toxicity in in vitro models and its levels are increased in human iPSCs, human postmortem putamen samples, and in striatal synaptosomes from mouse models of the disease. Here, we investigated the role of RTP801 in the hippocampal pathophysiology of HD. We found that RTP801 levels are increased in the hippocampus of HD patients in correlation with gliosis markers. Although RTP801 expression is not altered in the hippocampus of the R6/1 mouse model of HD, neuronal RTP801 silencing in the dorsal hippocampus with shRNA containing AAV particles ameliorates cognitive alterations. This recovery is associated with a partial rescue of synaptic markers and with a reduction in inflammatory events, especially microgliosis. Altogether, our results indicate that RTP801 could be a marker of hippocampal neuroinflammation in HD patients and a promising therapeutic target of the disease.

## 1. Introduction

Huntington’s disease (HD) is an incurable autosomal-dominant genetic disease caused by an abnormal expansion (of variable number) of CAG repeats in exon 1 of the *HTT* gene, which encodes for the huntingtin protein (htt) [1,2]. This expansion confers toxic properties to mutant htt (mhtt), leading to protein misfolding and aberrant aggregation. Although mhtt is the main contributor to the pathogenesis in HD, in recent years the CAG-expanded in the *HTT* gene was also identified as a toxic element in the disease at RNA level [3,4].

HD is characterized by a triad of motor, cognitive, and psychiatric symptoms [5,6,7]. Although the striatum is the most vulnerable brain area to synapse and neuronal degeneration [8,9], other regions such as cortex, cerebellum, and hippocampus are also affected [9,10,11,12]. Clinical manifestations of unequivocal motor alterations are generally a key factor for HD diagnosis, but subtle psychiatric and cognitive symptoms appear decades before the onset of motor dysfunction [5,13,14]. These symptoms include impairment in executive functions, attention, cognitive flexibility and learning, and memory, among others [13,14].

Several hippocampal alterations are also observed at pre-symptomatic stages in HD mouse models [15], including a reduction of synaptic markers [16,17,18,19] and structural abnormalities in neurons [15,18,20,21,22,23]. Moreover, increasing evidence highlights the relevant role of glial cells in the pathogenesis of HD (reviewed in [24,25]). Indeed, *HTT* is highly expressed in immune cells [26] and reactive microglia and astrocytes have been found in compromised brain areas in HD patients [8,27] and mouse models [16,28]. Interestingly, increased levels of both glial cell types and inflammatory mediators correlate with disease progression [24,27]. Hence, neuronal mhtt and astro-and micro-gliosis emerge as key contributors to HD pathogenesis.

Recent findings uncovered RTP801 protein as a crucial player in synapse pathology in neurodegenerative diseases, including HD [29]. REDD1/RTP801 protein is the coding product of the stress-induced gene *DDIT4* [30]. RTP801 mediates synaptic transmission and motor learning behavior in physiological conditions. Moreover, RTP801 abrogation increases GluA1 and TrkB levels [31]. RTP801 protein levels are also elevated in Parkinson’s disease (PD) [32,33], major depression [34] and Alzheimer’s disease (AD) [35]. Interestingly, RTP801 gene, *DDIT4*, is one of the top three common upregulated transcripts in postmortem brains from HD and PD patients [36]. In PD, RTP801 is sufficient to trigger neuronal death by sequentially inactivating Akt and mTOR [37,38]. Regarding HD, RTP801 mediates mhtt toxicity in cellular models and is upregulated in iPSCs derived from HD patients, in the putamen and cerebellum from HD patients [39] and in the striatum from mouse models. Moreover, in the R6/1 HD mouse model, striatal RTP801 silencing prevents motor learning deficits correcting, in turn, synaptic alterations by restoring the levels of GluA1 and TrkB [29,39].

In addition, we recently described that RTP801 is a crucial contributor to neuroinflammation and memory impairments in AD since its downregulation in hippocampal neurons prevents cognitive deficits in the 5×FAD mouse model and reduces inflammatory markers [35].

Although the therapeutic potential of RTP801 in motor dysfunction in HD has already been studied [29], the role of RTP801 in hippocampal cognitive impairment in the disease has not yet been addressed.

Here in this work, we investigated whether RTP801 downregulation is beneficial in a context of hippocampal dysfunction and the associated cognitive decline in HD. We found that in vivo RTP801 silencing in hippocampal neurons abrogates cognitive alterations and reduces the inflammatory response, including gliosis, in the R6/1 mouse.

## 2. Materials and Methods

### 2.1. Human Postmortem Samples

Postmortem hippocampal samples from HD patients and non-affected individuals were obtained from Neurological Tissue Bank of the Biobank-Hospital Clínic-IDIBAPS (IDIBAPS, Barcelona, Spain). Donation and obtention of samples were regulated by the guidelines and approval of the local ethics committee (Hospital Clínic of Barcelona’s Clinical Research Ethics Committee). The sample processing followed the rules of the European Consortium of Nervous Tissues: BrainNet Europe II (BNEII). All the samples were protected in terms of individual donor identification following the BNEII laws. Case information can be found in the Supplementary Materials Table S1. All the procedures for the obtention of postmortem samples followed the ethical guidelines of the Declaration of Helsinki and local ethical committees (Universitat de Barcelona ethical committee: IRB00003099).

### 2.2. Animals

As an HD model in this study, we used the transgenic mouse line R6/1 (RRID:IMSR_JAX:006471) maintained in a B6CBA background. Heterozygous R6/1 mouse expresses exon 1 of human mutant huntingtin (mhtt) with 115 CAG repeats, which codes for part of the N-terminal region of the protein, including the polyglutamine stretch. Transgene expression is driven by the human htt promoter. Wild type (WT) littermate animals were used as the control group. Experimental animals were all males and used at 8 weeks of age. All procedures were carried out in accordance with the National Institutes of Health Guide for the Care and Use of Laboratory Animals and approved by the local animal care committee of the Universitat de Barcelona (315/18 P10), following European (2010/63/UE) and Generalitat de Catalunya (10141-P10) regulations.

Mice were housed under controlled conditions (22 °C, 40–60% humidity in a 12 h light/dark cycle) with water and food available ad libitum. Mice were euthanized by cervical dislocation. The left hemisphere was dissected out for biochemical analyses while the right hemisphere was used for immunofluorescence techniques.

### 2.3. Tissue Fixation and Immunofluorescence

For immunofluorescence, dissected hemispheres were maintained in 4% PFA for 72 h and cryopreserved following a sucrose gradient (15% and 30%, 24 h each). Free-floating brain sections (30 µm) were obtained with a cryostat. Sections were incubated for 30 min in 50 mM NH_4_Cl to block autofluorescence. Blocking and permeabilization were performed for 1 h in blocking buffer (BB): PBS–T (Phosphate-buffered saline with 0.1% Tween-20) with 0.02% azide, 3% NGS, and 0.2% BSA (all from Thermo Fisher Scientific, Waltham, MA, USA). Primary antibodies were diluted in BB and incubated overnight at 4 °C in agitation. Secondary antibodies were diluted in BB and incubated for 2 h at room temperature. Nuclei were next stained with Hoechst 33342 (1:5000, Thermo Fisher Scientific, #H3570) in PBS for 15 min. Sections were washed twice in PBS–T between the different steps and a final wash with PBS was performed prior to mounting with ProLong Gold Antifade Mountant. Images were obtained with confocal microscopy (Zeiss LSM 880 and ZEN Software) at the advanced microscopy unit (Scientific and Technological Centers, University of Barcelona) with 10×, 25×, or 40× magnification and standard (1 airy disc) pinhole (1AU). Two sections from the dorsal hippocampus were analyzed per animal. The following primary antibodies were used: chicken polyclonal anti-GFP (1:1000, Synaptic Systems, Göttingen, Germany #132006), rabbit polyclonal anti-GFAP (1:500, Dako, Santa Clara, CA, USA, #GA52461), mouse monoclonal anti-GFAP (1:500, Sigma, Saint Louis, MO, USA, #G3893), rabbit polyclonal anti-Iba1 (1:500, Wako, Osaka, Japan, #09-19741), and rabbit polyclonal anti-RTP801 (1:100, Proteintech, Manchester, UK, #10638-1AP). The following secondary antibodies were used (all from Thermo Fisher Scientific): goat anti-chicken AlexaFluor488 (1:500, #A11039), goat anti-mouse AlexaFluor555 (1:200, #A21424), goat anti-mouse AlexaFluor647 (1:200, #A21236), goat anti-rabbit AlexaFluor555 (1:200, #A21430), and goat anti-rabbit AlexaFluor647 (1:200, #A21245).

### 2.4. Immunofluorescence Imaging and Analysis

Immunostained tissue sections were obtained by using a Zeiss LSM 880 (Carl Zeiss Microscopy, LLC, Thornwood, NY, USA) confocal microscope using the ZEN acquisition software. Images were obtained with a 25× magnification and standard (1 airy disc) pinhole (1AU). Images were analyzed with ImageJ software (NIH, Bethesda, Montgomery, AL, USA). For GFAP and Iba1 staining, the same threshold was applied to properly select individual glial cells. A mask was created to individually measure the mean staining intensity in each cell. For RTP801 staining measurements in astrocytes, RTP801 mean intensity was quantified within the same mask used for GFAP staining analysis. Two different sections of the dorsal hippocampus were measured for each animal, where the mean intensity from all detected cells was calculated for each image. Histograms show the mean intensity of the two images per animal [16,40].

### 2.5. Western Blotting

For naïve mice, both hippocampi were dissected out and homogenized together. For mice undergoing surgeries, the dorsal and ventral hippocampus from the left hemisphere were dissected out separately. Crude synaptosomal fractions were obtained as described elsewhere [35]. Protein concentration of all samples was established with Bradford reagent (Bio-Rad, Hercules, CA, USA) following the manufacturer’s instructions. Protein samples (15–20 µg) were prepared with Pierce TM Lane Marker reducing sample buffer and heated at 96 °C for 5 min. Samples were resolved in NuPAGE^TM^ Novex^TM^ polyacrylamide gels. Then, 3–8% polyacrylamide gels with Tris-Acetate running buffer were used to analyze proteins with high molecular weight, while 12% and 4–12% polyacrylamide gels with MOPS SDS running buffer were used for proteins with small and intermediate weights, respectively. The molecular weight marker used was the PageRuler Pre-stained protein ladder and gels were run in the XCell SureLock Mini-Cell system. Proteins were transferred to nitrocellulose membranes with the iBlot system. All reagents and machinery were obtained from Thermo Fisher Scientific. Next, membranes were blocked with 5% non-fat dry milk (Bio-Rad) diluted in TBS-T (Tris-buffered saline 0.1% Tween-20) for one hour. Primary antibodies were diluted in TBS-T and 5% BSA and incubated overnight at 4 °C in agitation. HRP-conjugated anti-β-actin primary antibody was incubated for 30 min before chemiluminescent protein detection. HRP-conjugated antibodies were diluted 1:10,000 in TBS-T and 5% non-fat dry milk for 1 h at room temperature.

Primary antibodies used were (1:1000 if not stated otherwise): rabbit polyclonal anti-GFAP (Dako, #GA52461), chicken polyclonal anti-GFP (Thermo Fisher Scientific, #A-11122), rabbit polyclonal anti-RTP801 (1:500, Proteintech, #10638-1-AP for murine samples and 1:500, FineTest, #07228 for human samples), mouse monoclonal anti-GFAP (1:500, Sigma, #G3893), rabbit polyclonal anti-Iba1 (Wako, #019-19741), mouse monoclonal anti-TrkB (BD Biosciences, Franklin Lakes, NJ, USA, #610102), mouse monoclonal anti-PSD-95 (Thermo Fisher Scientific, #MA1-045), rabbit polyclonal anti-GluA1 (Merck, Waltham, MA, USA #ABN241), rabbit polyclonal anti-Akt P-S73 (Cell Signaling Technology, Danvers, MA, USA #376234), rabbit polyclonal anti-Total Akt (Cell Signaling Technology, #4691), rabbit polyclonal anti-mTOR P-S2448 (Cell Signaling Technology, #2971), rabbit polyclonal anti-mTOR (Cell Signaling Technology, #2971), rabbit monoclonal anti-S6 P-S235/236 (Cell Signaling Technology, #4858), rabbit polyclonal anti-S6 (Cell Signaling Technology, #4858), rabbit polyclonal anti-NRLP1 (1:1000, Novus, Centennial, CO, USA #NBP1-54899), rabbit monoclonal anti ASC/TMS1 (Cell Signaling Technology, 1:1000, #67824), rabbit monoclonal anti-Cleaved Caspase-1 (Cell Signaling Technology, 1:500, #89332), rabbit monoclonal anti-Caspase-1 (Cell Signaling Technology, 1:500, #24232), and anti-β-actin (1:100,000, Sigma, #A3854). HRP-conjugated goat anti-mouse or anti-rabbit IgG were diluted 1:10,000 (Thermo Fisher Scientific, #31430 and #31460, respectively). Proteins were detected with Supersignal^TM^ West Pico Plus chemiluminescent substrate (Thermo Fisher Scientific). Images were acquired with ChemiDoc^TM^ (Bio-Rad, Hercules, CA, USA) and quantified by densitometric analysis with ImageJ software (NIH). When re-incubation with another primary antibody was needed, membranes were washed with Restore Plus Western Blot Stripping buffer (Thermo Fisher Scientific, Waltham, MA, USA) for 15 min to remove the previous signal.

### 2.6. Hippocampal Injection of Adeno Associated (AAV) Viral Vectors

AAVs containing a scrambled shRNA (5′-GTGCGTTGCTAGTACCAAC-3′) as control or an shRNA against RTP801 (5′-AAGACTCCTCATACCTGGATG-3′) to knockdown RTP801 expression were applied in the hippocampus. Both sequences had been previously verified [29,37]. ShRNAs cloning and AAV viral particles were generated by the Unitat de Producció de Vectors from the Center of Animal Biotechnology and Gene Therapy at the Universitat Autònoma de Barcelona.

Following anesthesia with isoflurane (5% induction, 1.5% maintenance), mice were subjected to bilateral intrahippocampal injections of rAAV2/8-H1-shRTP801-RSV-GFP (1.07 × 10^13^ GCs) or a control rAAV2/8-H1-shControl-RSV-GFP (1.2 × 10^13^ GCs). Two injections were performed in the hippocampus in both hemispheres. The following coordinates relative to Bregma (anteroposterior and lateral) and from skull (dorsoventral) were used (in mm): anteroposterior, −2; mediolateral, +/−1.5; and dorsoventral, −2.1 (for DG) and −1.3 (for CA1). In each depth 1:1 virus (1 µL) was infused. Viral vectors were injected with a 10 µL Hamilton syringe at an infusion rate of 250 nL/min. The needle was left in place for 2 min to ensure complete diffusion of the AAVs. Mice were returned to their home cage after 1 h of careful monitoring. Behavioral assessment was performed 4 weeks after surgery.

### 2.7. Behavioral Assessment

Open field test: Mice were placed in a 40 cm × 40 cm × 40 cm arena with dim light for 30 min. The center area was considered as the central squared 20 × 20 cm space.

Spontaneous alternation in a T-maze: For this test, a T-shaped maze with three arms was used. Arms were 45 cm long, 20 cm high, and 8 cm wide and were separated by a central 10 cm wide square. In the acquisition phase, one of the arms was closed (called novel arm, randomized for genotype and shRNA) and mice were allowed to explore the maze for 10 min. Two hours later, both arms were opened and then mice were allowed to freely explore the maze for 5 min (retrieval phase). In both phases, mice were initially placed in the end of the central arm. First arm choice was considered when mice entered an arm with the two front limbs and was represented as alternation rate (%).

Passive avoidance task: For this test, we used a two-compartment box where both chambers were separated by a gate (5 cm × 5 cm). One compartment was dimly lit (20 lx) while the other was brightly lit (200 lx). On the first day, (training) mice were placed in the brightly lit compartment with the gate closed for 10 s. Next, the gate was open, and mice were allowed to enter the dark compartment. Latency to cross (4 paws inside the dark chamber) was measured. Once mice entered the dark area, a 4.6 mA foot shock was given for 2 s. Mice were kept in the dark compartment for 20 s before being returned to their home cage. On testing days, mice were placed in the brightly lit compartment with the gate open. Latency to enter the shock-paired compartment was measured for a maximum of 10 min (600 s cutoff). After crossing the gate mice were kept in the dark compartment for 20 s before being returned to their home cage. Mice were forced to enter the dark compartment after the 600 s cutoff.

Mice movement was tracked and recorded using SMART 3.0 Software (Panlab, Barcelona, Spain). Other parameters were manually monitored. Experiments were performed in a blind-coded manner with respect to genotype and shRNA. Mice handling procedures were performed once a day for 5 days, prior to behavioral testing. Mice were allowed to habituate to the experimental room in their home cages for 1 h before behavioral sessions. Behavioral objects and apparatus were always cleaned with water and dried between animals to avoid olfactory interferences.

### 2.8. Statistics

Sample sizes were determined by using the power analysis method: 0.05 alpha value, 1 estimated sigma value, and 75% of power detection. All data are expressed as mean ± SEM. Normal distribution was tested with d’Agostino and Pearson omnibus, Shapiro–Wild and Kolmogorov–Smirnov normality tests. If the test was valid, parametric statistical analyses were performed. Before pairs of comparisons, we performed the F test to compare variances. In experiments with normal distribution, statistical analyses were performed using the unpaired two-sided Student’s *t*-test (95% confidence) and the two-way ANOVA with Bonferroni’s post hoc tests as appropriate (indicated in the figure legends). A t-test with Welch’s correction was applied when variances were unequal. Values of *p* < 0.05 were considered statistically significant. Chi-square (χ^2^) test was performed in pair comparisons. Correlation analyses were performed using Pearson. Grubbs’ and ROUT tests were performed to determine the significant outlier values. All experiments in this study were blinded and randomized by blocks of animals. All mice bred for the experiments were used for pre-planned experiments and randomized to experimental groups. Data were collected, processed, and analyzed randomly. The experimental design and handling of mice were identical across experiments. Littermates were used as controls with multiple litters (3–5) examined per experiment.

## 3. Results

### 3.1. RTP801 Levels Are Increased in the Hippocampus from HD Patients and Correlate with Neuroinflammatory Markers

**Figure 1:**
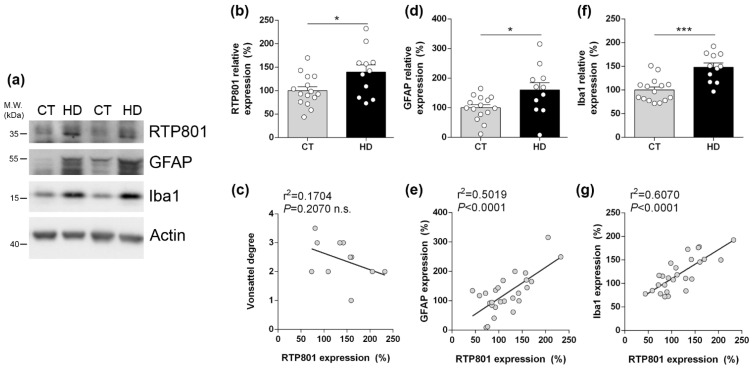

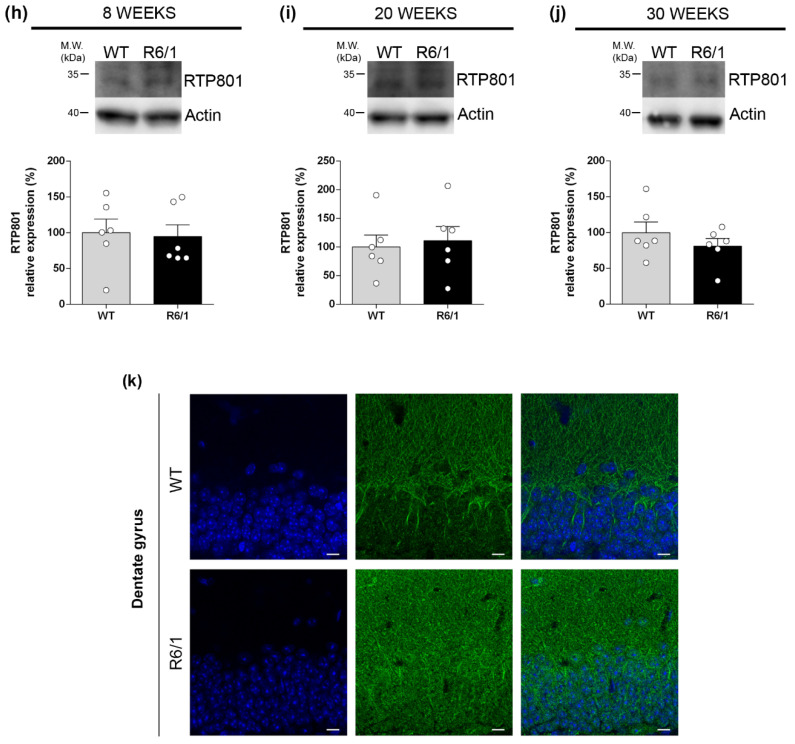
RTP801 levels are increased in the hippocampus of HD patients and correlate with neuroinflammatory markers but are not altered in the R6/1 mouse. (**a**) Immunoblotting for RTP801, GFAP, Iba1, and actin as loading control in total homogenates from human post-mortem hippocampal samples from non-affected individuals (CT) and Huntington’s disease (HD) patients. Densitometric quantifications of RTP801 (**b**), astrogliosis marker GFAP (**d**), and microgliosis marker Iba1 (**f**). Pearson’s correlation analysis comparing RTP801 levels as in (**b**) with Vonsattel grades (**c**), GFAP levels (**e**), and with Iba1 levels (**g**) per sample. (**h**–**j**) Immunoblotting for RTP801 and actin as loading control in total homogenates from 8, 20, and 30 weeks old wild-type (WT) and R6/1 mice. (**i**) Densitometric quantification of RTP801 protein levels. Data in (**b,d,f,i**) is represented as mean ± SEM and was analyzed with Student’s *t*-test. * *p* < 0.05, *** *p* < 0.001 vs. CT. (**k**) RTP801 immunostaining in 8-week-old WT and R6/1 mouse hippocampus (dentate gyrus). Coronal sections were stained with an antibody against RTP801 (in green) and nuclei were visualized with Hoechst 33352 staining (in blue). Scale bars, 10 µm.

Previous results from our lab showed that RTP801 levels are increased in brain homogenates obtained from HD patients putamen and cerebellum [29,39]. Here, in human post-mortem hippocampal lysates, we found that RTP801 protein levels were significantly increased in HD patients compared with controls (Figure 1a,b). No significant correlation was found between RTP801 levels and Vonsattel grades (Figure 1c). GFAP (a marker of astrocytes) and Iba1 (a marker of microglia) levels were increased in HD patients (Figure 1d,f) and positively correlated with RTP801 levels (Figure 1e,g), further suggesting that RTP801 levels could be a marker of gliosis in HD [35].

Altered RTP801 levels were previously detected in striatal samples from the Hdh^Q7/Q111^ and R6/1 HD mouse models [29]. Here, we investigated whether hippocampal RTP801 levels were altered during the progression of the disease in R6/1 mice: presymptomatic (8 weeks of age) and when they already display motor dysfunction and memory impairments (20 and 30 weeks of age) [41,42]. No differences in total hippocampal RTP801 levels between WT and R6/1 mouse were detected (Figure 1h–j). However, RTP801 staining pattern is altered in the hippocampus of the R6/1 model, suggesting a differential intraneuronal redistribution (Figure 1k).

### 3.2. RTP801 Silencing in Hippocampal Neurons Prevents Cognitive Dysfunction in the R6/1 Mouse

Evidence showed that RTP801 silencing in several mouse models of neurodegenerative diseases ameliorates their pathologic phenotype. Hence, although RTP801 levels were not significantly increased in the R6/1 mouse hippocampus, we genetically downregulated neuronal hippocampal RTP801 levels in 8-week-old WT and R6/1 mice. At this age, the severe hippocampal-dependent memory deficits have not appeared yet [19,43]. Four groups of mice were generated: WT shCt, WT shRTP801, R6/1 shCt, and R6/1 shRTP801.

**Figure 2:**
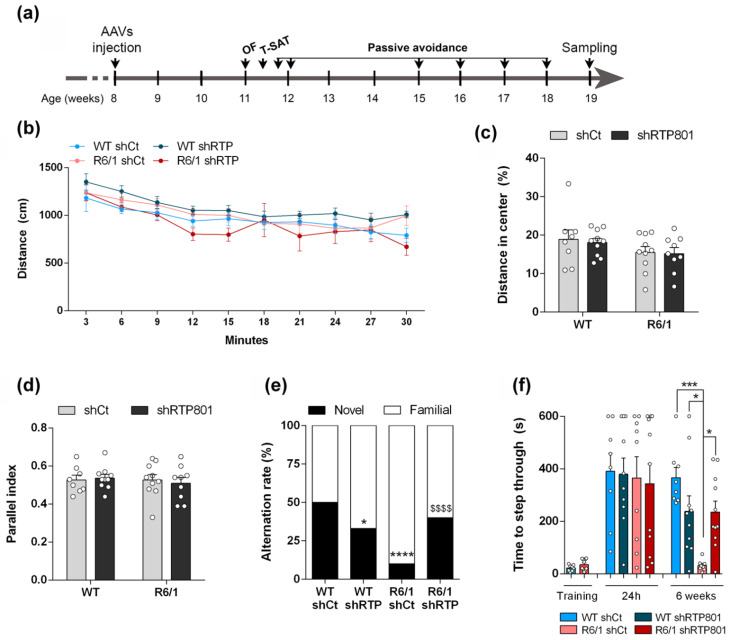
Silencing hippocampal RTP801 levels prevents cognitive dysfunction in the R6/1 mouse. (**a**) AAVs expressing GFP-shCT (AAV-shCt) or GFP-shRNA-RTP801 (AAV-shRTP801) were bilaterally injected in the dorsal hippocampus of 2-month-old WT and R6/1 male mice. A battery of behavioral tests was performed 4 weeks later. (**b**–**d**) Locomotor activity in the open field was assessed for 30 min. Total distance travelled each 3 min (**b**) (two-way ANOVA group effect for travelled distance F_(3,32)_ = 1.767, *p* = 0.1732), distance travelled in the center (**c**) (treatment effect: F_(1, 33)_ = 0.1085, *p* = 0.7439; genotype effect: F_(1, 33)_ = 3.517, *p* = 0.0696), as a measure of anxiety, and parallel index (**d**) (treatment effect: F_(1, 33)_ = 0.02069, *p* = 0.8865; genotype effect: F_(1, 33)_ = 0.2781, *p* = 0.6015) were monitored. No differences were found between groups. Data are represented as mean ± SEM and were analyzed with two-way ANOVA. (**e**) Spontaneous alternation rate 2 h after the training trial was assessed in the T-SAT. Chi-square (χ^2^) test was performed in pair comparisons: * *p* < 0.05 and **** *p* < 0.0001 compared with WT shCt and ^$$$$^ *p* < 0.0001 compared with R6/1 shCt. (**f**) In the passive avoidance test, the latency (in seconds) to step-through was measured before (training), 24 h after and weekly after training (6 weeks of data are shown in the graph). Data from the 6-week testing day was analyzed with one-way ANOVA (F = 8.542, *p* = 0.0002) followed by Bonferroni’s post hoc test: * *p* < 0.05 and *** *p* < 0.001. Data are means ± SEM.

Four weeks after AAVs injection, when cognitive symptoms had already appeared in this mouse model [42,43], we performed a broad behavioral characterization as depicted in Figure 2a. First, all mice were subjected to the open field test (OF) to discard a shRTP801 effect over general locomotion, since hippocampal principal neurons can regulate motor behavior [44]. RTP801 downregulation did not induce significant changes in travelled distance, thigmotaxis, or parallel index (Figure 2b–d). No differences were found between genotypes, as expected [45] (Two-way ANOVA group effect for travelled distance F_(3,32)_ = 1.767, *p* = 0.1732; genotype effect for percentage of distance in the center of the arena F_(1, 33)_ = 3.517, *p* = 0.0696; and genotype effect for parallel index F_(1, 33)_ = 3.712, *p* = 0.0627).

Next, we analyzed spontaneous alternation in a T-maze task, 2 h after habituation. We observed reduced cognitive flexibility in the R6/1 shCt group, since they showed no tendency to modify their response and explore the novel arm. This parameter was rescued in shRTP801-injected R6/1 mice to levels similar to WT shCt and WT shRTP801 groups (Figure 2e) (χ^2^:_2.020_, *p* = 0.1552 vs. WT shCt and χ^2^:_1.057_, *p* = 0.3039 vs. WT shRTP801).

The passive avoidance paradigm is a hippocampal-related test used to evaluate learning and memory [46,47]. In this test, we did not observe significant differences in the step-through latencies between groups in the first retention test, 24 h after training (one-way ANOVA F_(3,34)_ = 0.08203, *p* = 0.9694) (Figure 2f, 24 h testing session) meaning none of the groups showed associative memory deficits at this time point. However, 6 weeks later, shCt-injected R6/1 mice showed a significant reduction of the acquired long-lasting associative memory. In contrast, the WT shCt, WT shRTP801, and R6/1 shRTP801 groups showed similar levels of aversion to the dark chamber than in the first retention test (Figure 2f, 6 weeks session). Thus, RTP801 silencing in hippocampal neurons prevented cognitive alterations in terms of spatial working memory and long-lasting associative memory.

### 3.3. Silencing RTP801 Levels in R6/1 Mouse Hippocampal Neurons Prevents Neuroinflammatory Processes

**Figure 3:**
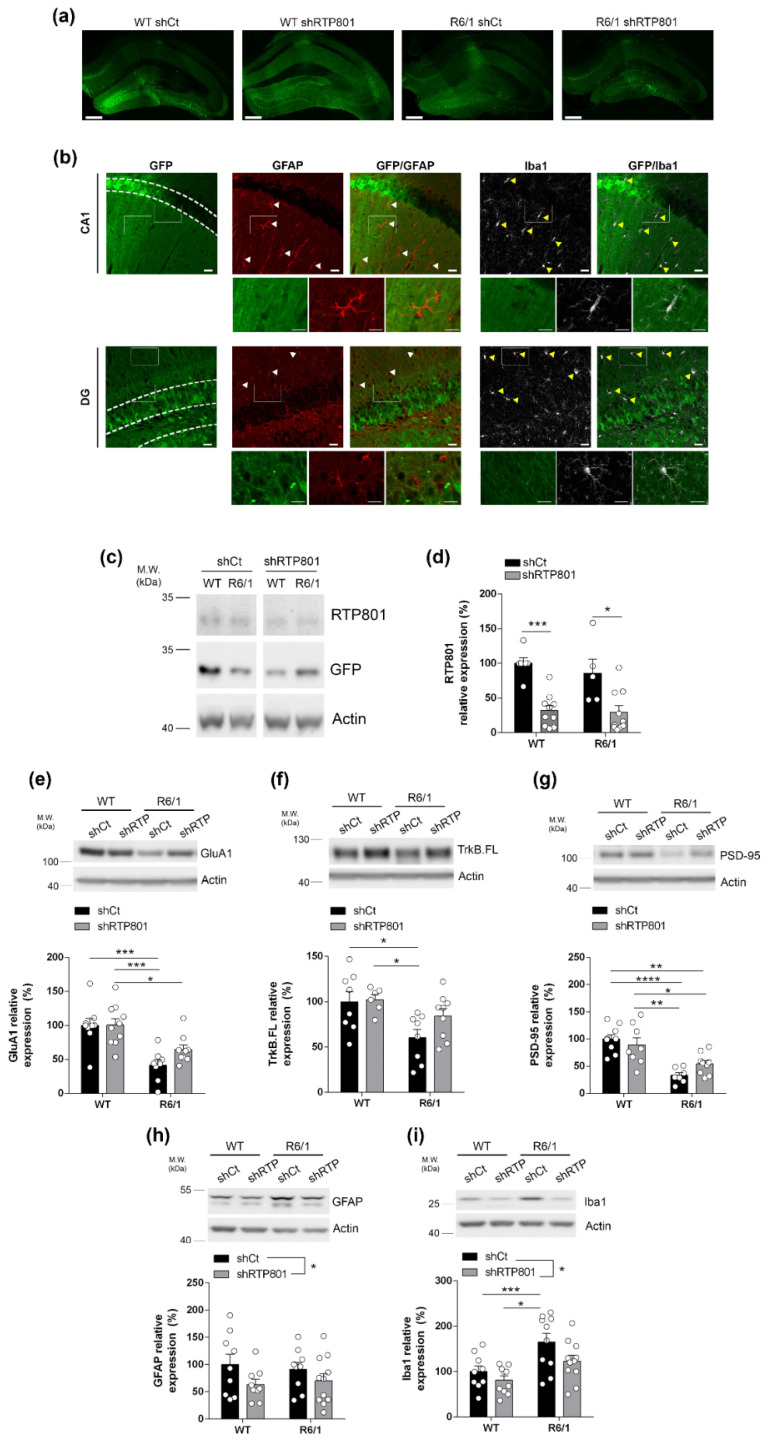
RTP801 hippocampal silencing is restricted to principal neurons, partially restores synaptic GluA1 and TrkB.FL levels, and reduces neuroinflammatory markers in the R6/1 mouse. (**a**) Representative dorsal hippocampi from WT shCt, WT shRTP801, R6/1 shCt, and R6/1 shRTP801 mice 11 weeks after the injection; GFP fluorescence (green) was detected in the entire dorsal hippocampus. Scale bars, 250 µm. (**b**) Neither astrocytes (stained against GFAP, red) nor microglial cells (Iba1 + cells, grey) express GFP protein, indicating they were not transduced with AAV particles. CA1 and DG are depicted through dashed lines. Arrowheads indicate non-transduced glial cells. White rectangles in upper panels show digitally zoomed images (lower panels) to depict specific cells. Scale bars, 50 and 15 µm. (**c**) Representative immunoblot showing the levels of RTP801 relativized with respect to GFP/actin ratio levels as the corresponding loading controls in dorsal hippocampus extracts. (**d**) The histogram represents protein levels expressed as percentage of WT shCt (treatment effect: F_(1, 27)_ = 31.18, *p* < 0.0001; genotype effect: F_(1, 27)_ = 0.5064, *p* = 0.4828). (**e**–**g**) Representative immunoblots showing the levels of GluA1 (treatment effect: F_(1, 32)_ = 1.771, *p* = 0.1927; genotype effect: F_(1, 32)_ = 27.79, *p* < 0.0001), TrkB.FL (treatment effect: F_(1, 27)_ = 1.954, *p* = 0.1736; genotype effect: F_(1, 27)_ = 9.614, *p* = 0.0045) and PSD-95 (treatment effect: F_(1, 29)_ = 0.3480, *p* = 0.5598; genotype effect: F_(1, 29)_ = 32.94, *p* < 0.0001) normalized by actin in the crude synaptosomal fraction obtained from dorsal hippocampus extracts from WT shCt, WT shRTP801, R6/1 shCt, and R6/1 shRTP801 groups of mice. (**h**,**i**) Representative immunoblots showing the levels of GFAP (treatment effect: F_(1, 34)_ = 4.222, *p* = 0.0477; genotype effect: F_(1, 34)_ = 0.004228, *p* = 0.9485) and Iba1 (treatment effect: F_(1, 36)_ = 4.691, *p* = 0.0370; genotype effect: F_(1, 36)_ = 14.22, *p* = 0.006) normalized by actin in total homogenates obtained from dorsal hippocampus extracts from WT shCt, WT shRTP801, R6/1 shCt, and R6/1 shRTP801 groups of mice. Data are means ± SEM and were analyzed by two-way ANOVA followed by Bonferroni’s post hoc test. * *p* < 0.05, ** *p* < 0.01, and *** *p* < 0.001, **** *p* < 0.0001 compared with WT shCt.

One week after finishing the battery of behavioral testing, the brains were collected to investigate the events that could explain the affectation of the hippocampal-dependent cognitive behavior. We first confirmed a widespread viral transduction in the dorsal hippocampus including the dentate gyrus (DG) and the CA1 (Figure 3a). We also confirmed that principal neurons (pyramidal neurons in the CA1 and granule cells in the DG) but not glial cells were transduced with the neuron specific AAV2/8 particles (Figure 3b and Supplementary Materials Figure S1). Downregulation of RTP801 levels was confirmed by Western blot in samples from the dorsal hippocampus (Figure 3c,d).

Previous findings described that hippocampal neuropathology in R6/1 mice is accompanied by reduced levels of synaptic markers such as PSD-95, AMPAR subunit GluA1, and full-length BDNF receptor TrkB (TrkB.FL) [18,21,48,49]. Interestingly, we observed that silencing RTP801 in the R6/1 mouse hippocampus partially rescued GluA1 and TrkB.FL receptors levels (Figure 3e,f) by a mTOR-independent mechanism (Supplementary Materials Figure S2) although it did not recover the loss of PSD-95 (Figure 3g) levels.

Since RTP801 levels strongly correlate with gliosis markers in HD human samples (Figure 1e,g) and the protein downregulation was associated with an anti-inflammatory effect in a mouse model of AD, we next investigated whether RTP801 silencing was affecting core neuroinflammatory events in the R6/1 model. Indeed, silencing neuronal RTP801 in both WT and R6/1 mice reduced significantly GFAP and Iba1 levels (Figure 3h,i). While astrogliosis was not observed in R6/1-shCt mice, increased levels of Iba1, as a readout for microgliosis, were detected.

**Figure 4:**
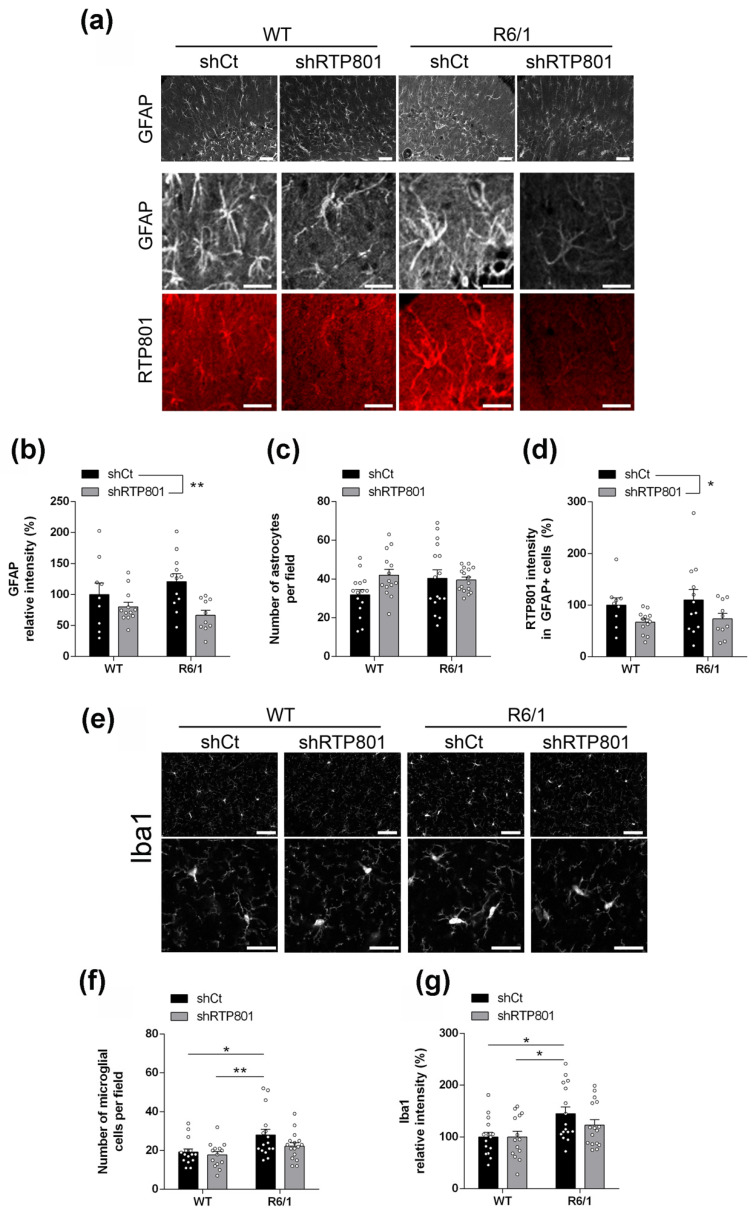
Silencing hippocampal RTP801 neuronal levels in R6/1 mouse partially prevents neuroinflammatory events. (**a**) Representative GFAP and RTP801 labelling in the CA1 hippocampal region in 5-month-old WT and R6/1 mice (upper panels, scale bars 50 µm and lower panels, 25 µm). (**b**) Quantification of GFAP levels (IOD intensity, % respect to WT shCt) in the four groups (treatment effect: F_(1, 39)_ = 9.363, *p* = 0.0040; genotype effect: F_(1, 39)_ = 0.09983, p = 0.7537). (**c**) Quantification of GFAP-positive cell density in the four groups (treatment effect: F_(1, 57)_ = 2.271, *p* = 0.1373; genotype effect: F_(1, 57)_ = 0.9986, *p* = 0.3219). (**d**) Quantification of RTP801 levels in GFAP-positive cells in the four groups (treatment effect: F_(1, 39)_ = 5.909, *p* = 0.0198; genotype effect: F_(1, 39)_ = 0.3525, *p* = 0.5562). (**e**) Representative Iba1 labelling from the CA1 in 5-month-old animals (upper panels, scale bars 50 µm and lower panels, 25 µm). (**f**) Quantification of Iba1-positive cell density in the CA1 in the four groups (treatment effect: F_(1, 57)_ = 3.304, *p* = 0.0744; genotype effect: F_(1, 57)_ = 9.952, *p* = 0.0026). (**g**) Quantification of Iba1 relative intensity (% respect to WT shCt) in the CA1 in the four groups (treatment effect: F_(1, 56)_ = 1.377, *p* = 0.2456; genotype effect: F_(1, 56)_ = 10.13, *p* = 0.0024). Immunolabelling quantification of all proteins is expressed as the mean ± SEM. All data were analyzed by two-way ANOVA followed by Bonferroni’s post hoc test. * *p* < 0.05, and ** *p* < 0.01.

We confirmed the abovementioned results by immunofluorescence. Knocking down RTP801 in neurons reduced GFAP-immunoreactivity in both WT and R6/1 mice hippocampus (Figure 4a,b). No significant effect was found in the number of astrocytes (Figure 4a,c) but, interestingly, RTP801 expression in astrocytes was reduced (Figure 4a,d), although this cell type was not transduced with the neuron-specific AAVs.

Regarding microglia, we detected a mild recovery in both Iba1-immunoreactivity and microglia density in R6/1-shRTP801 mice (Figure 4e–g). We also checked for morphological changes in microglia associated with an inflammatory response, but no genotype or shRNA effects were observed (Supplementary Materials Figure S3) as previously described in the striatum of R6/1 mouse at this age [50].

**Figure 5:**
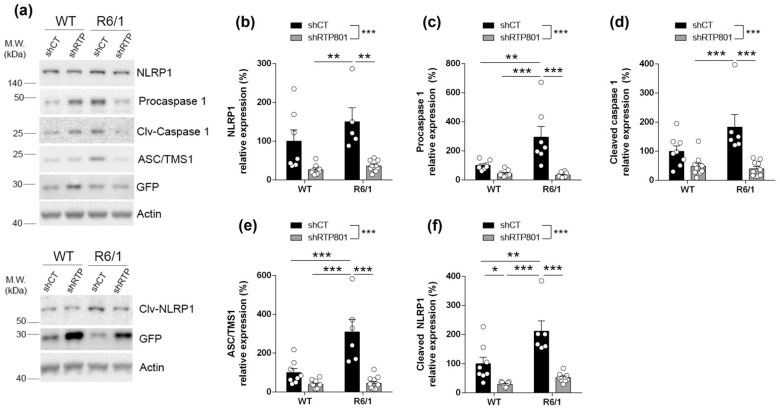
RTP801 silencing in the R6/1 mouse hippocampal neurons reduces the levels of the inflammasome components. (**a**) Immunoblottings for NLRP1, cleaved NLRP1, procaspase 1, cleaved caspase 1, ASC/TMS1, and GFP as loading control for transduced neurons in the dorsal hippocampus of 4.5-month-old WT shCt, WT shRTP801, R6/1 shCt, and R6/1 shRTP801 mice. (**b**–**f**) Densitometric quantification of NRLP1 (treatment effect: *F*_(1, 26)_  = 24.89, *p*  < 0.0001; interaction: *F*_(1, 26)_  =  1.175, *p*  =  0.2883), cleaved NLRP1 (treatment effect: *F*_(1, 29)_  =  44.18, *p* < 0.0001; genotype effect: *F*_(1, 29)_  =  15.32, *p* = 0.0005; interaction: *F*_(1, 29)_  =  6.588, *P*  =  0.0157), ASC/TMS1 (treatment effect: *F*_(1, 31)_  =  40.72, *p* < 0.0001; genotype effect: *F*_(1, 31)_  =  17.85, *p* = 0.0002; interaction: *F*_(1, 31)_  =  15.91, *p*  =  0.0004), procaspase 1 (treatment effect: *F*_(1, 30)_  = 26.06, *p*   < 0.0001; interaction: *F*_(1, 30)_  =  10.82, *p*  =  0.0026) and cleaved caspase 1 (treatment effect: *F*_(1, 30)_  = 25.57, *p*   < 0.0001; interaction: *F*_(1, 30)_  =  5.551, *p*  =  0.0252) as in (**a**) for the hippocampus. Densitometric quantification of all proteins is expressed as the mean  ±  SEM. All data were analyzed by two-way ANOVA followed by Bonferroni’s post hoc test. * *p*  <  0.05, ** *p*  <  0.01, and *** *p*  <  0.001.

To understand the mechanism by which silencing RTP801 in hippocampal neurons diminished the inflammatory response in the R6/1 mice, we investigated whether the inflammasome receptor NLRP1 and its components were affected (Figure 5a). NLRP1 is mainly expressed in neurons [51] as pro-form and auto-proteolytic fragment (cleaved). Silencing neuronal RTP801 diminished the activation of NLRP1 (Figure 5b,c) in both WT and R6/1 mice. Moreover, this significant reduction was accompanied by a reduction of the levels of the procaspase 1 (Figure 5e), its cleaved form (Figure 5f), and the inflammasome adaptor ASC/TMS1 (Figure 5d).

Altogether, our results suggest that neuronal hippocampal RTP801 in the R6/1 mouse model affects cognition by impairing the expression of synaptic proteins and activating the inflammasome that will eventually induce astro- and microglial reactivity.

## 4. Discussion

Here, we found that neuronal RTP801 is involved in hippocampal pathophysiology in HD as its RTP801 downregulation in the R6/1 mouse prevented some cognitive alterations, partially restored the levels of synaptic GluA1 and TrkB.FL receptors, and reduced inflammasome activation and gliosis.

Our previous studies demonstrated the contribution of striatal RTP801 to HD pathology. RTP801 mediates mhtt toxicity in vitro [39] and its levels appear increased in cortical and putamen/striatal samples from HD patients and mouse models of the disease [29,39]. However, the putative role of RTP801 in HD hippocampal pathology had never been investigated. Previous works have addressed the contribution of the protein to hippocampal function. For instance, in WT animals, RTP801 overexpression impairs contextual-fear memory consolidation, while RTP801 hippocampal silencing enhances this process [52]. In line with this, RTP801 hippocampal downregulation prevents memory impairments and reduces neuroinflammation in the 5xFAD mouse model of AD [35] and diminishes amyloid-beta-induced synaptic dysfunction [53].

Here, we found that RTP801 protein is upregulated in postmortem hippocampal samples from human HD patients, an increase that significantly correlated with the gliosis markers GFAP and Iba1, in line with our previous study in AD (ref paper AD). Future studies should address the potential association of RTP801 expression with the Vonsattel neuropathological degrees and the size of the HTT expansion, since our study does not include samples with the highest neuropathological grading or high number of CAG repeats.

Then, we reduced RTP801 levels with neuron specific AAVs (serotype 2/8) in the dorsal hippocampus in the R6/1 model of HD. As stated in our previous work [29] and in accordance with the lack of neuronal death in HD mouse models [41,48], hippocampal levels of RTP801 in homogenates were not altered in R6/1 mouse. However, we cannot exclude the speculation that mhtt leads to an altered distribution in neurites or spines rather than differences in total RTP801 levels that could contribute to plasticity impairment in the pathology. Nevertheless, our experiments confirmed the previous studies indicating that R6/1 mice display impaired memory and cognitive flexibility, features which are also affected in human HD patients [15,49]. RTP801 silencing in hippocampal neurons with a validated shRNA against RTP801 [29,32,33,37,39,54] prevented the alterations in cognitive flexibility and long-lasting associative memory observed in shCt-R6/1 mice, while no effects were found in WT animals. We speculate that, although the levels of hippocampal RTP801 do not differ between WT and R6/1 animals, the presence of mhtt could be influencing RTP801 detrimental effects in neurons, explaining the beneficial outcome of silencing it.

We also confirmed the decreased levels of synaptic markers such as PSD-95, GluA1 AMPAR subunit, and TrkB.FL receptor in the R6/1 mouse hippocampus [18,48,49]. Remarkably, a mild restoration of GluA1 and TrkB.FL synaptic levels was observed, in line with previous results from our group [29,31]. Indeed, the RTP801 total knockout mouse shows increased levels of cortical GluA1 and TrkB.FL, in good correlation with the enhanced synaptic transmission observed in these animals [31], and RTP801 striatal silencing in the R6/1 model enhances the expression of both synaptic proteins in this brain area [29]. On the one hand, AMPAR subunit GluA1 confers calcium permeability to the receptor, promoting the activation of intracellular signaling cascades critical for synaptic transmission and plasticity [55,56,57,58]. On the other hand, increased TrkB.FL levels could promote neuronal survival, neuroprotection, hippocampal long-term potentiation and also GluA1 local translation [59,60]. These observations suggest that hippocampal RTP801 silencing could enhance synaptic transmission and neuroprotection which, in turn, would contribute to the prevention of cognitive abnormalities observed in the R6/1 mouse.

Remarkably, RTP801 silencing led to a general decrease in GFAP protein levels and a normalization of microgliosis in the dorsal hippocampus of the R6/1 mouse, as readouts of inflammation. We speculate that this reduction of neuroinflammatory markers was also contributing to the amelioration of cognitive deficits in the R6/1-shRTP801 group since in recent years increasing evidence indicate an important role of glial cells in learning and memory processes. For instance, astrocytes regulate memory processes [61,62,63] and have a key role in hippocampal synaptic plasticity [64]. Regarding microglia, this cell type also regulates synaptic plasticity by controlling synapses formation in the healthy and diseased brain [65] and its depletion leads to altered spatial learning [66]. Indeed, microglial replacement improves cognition in aged mice [67] and they can trigger synaptic transmission by the release of modulatory factors [68]. Moreover, several investigations indicate that the activation of inflammatory cells plays an important role in the pathogenesis of HD (reviewed in [24]). First, early studies described reactive gliosis in human HD brains [8,69,70,71] and, indeed, increased gliosis and inflammatory mediators correlate with disease progression [24,27]. Second, both astrocytes and microglia show a wide range of morphological and functional alterations in HD [25,50,72,73,74,75]. Finally, some therapeutic approaches proven effective in the amelioration of HD phenotype involved decreased gliosis in the striatum or hippocampus [16,50,76,77,78]. Here, we confirm that inflammatory processes are involved in hippocampal-related pathology in HD and suggest that modulation of this inflammatory response could serve to improve cognitive function.

Given that RTP801 downregulation was specifically in neurons, further research should be performed in a complementary cell-specific manner, to elucidate the importance of silencing RTP801 in glial cells. We speculate that the reduction of the activation of the inflammasome receptor NLRP1, mostly expressed in neurons [51], upon RTP801 silencing, would lead to a decrease in astrocytic RTP801 levels and would contribute to preventing the inflammatory response. In fact, silencing neuronal RTP801 also affected NLRP1 downstream effectors and adaptors, confirming this hypothesis. Hence, RTP801 silencing can reduce the activation of neuronal inflammatory pathways that trigger astrocyte and microglia reactivity in HD models.

Interestingly, increased RTP801 levels are also associated with the activation of inflammatory pathways in non-neuronal models [79,80,81]. Indeed, in macrophages exposed to lipopolysaccharide (LPS) or lung epithelium to cigarette smoke, RTP801 abrogation reduced the release of proinflammatory mediators and the inflammasome formation, among others [79,80,81]. Moreover, our previous work described a novel pathological mechanism for RTP801 in neuroinflammation in AD, which also involved reduced gliosis and decreased levels of NLRP1 receptor [35]. Hence, our data suggest a similar role of RTP801 in neuroinflammation in a context of HD.

Altogether, our results support RTP801 as a readout for hippocampal pathology in HD patients and highlight RTP801 downregulation as a promising therapeutic strategy to ameliorate inflammatory events and to prevent motor and cognitive deficits in Huntington’s disease.

## Data Availability

Not applicable.

## Supplementary Materials

The following are available online at https://www.mdpi.com/article/10.3390/biom12010034/s1, Figure S1: Immunostaining of 8-weeks-old WT mouse hippocampus. Coronal sections were stained with antibodies against GFP protein (in green) and MAP2 (in red) and images were taken under epifluorescence microscopy (scale bar is 20 microns in all images). Figure S2: mTOR activity in dorsal hippocampal samples from WT and R6/1 mice injected with AAV and Figure S3: Microglial morphology in WT and R6/1 mice injected with AAV in the hippocampus; Table S1: Neuropathological hallmarks are indicated: Vonsattel degrees, ranging from 1 to 4 and number of CAG repeats.
