# Supplementary material for: RTP801/REDD1 Is Involved in Neuroinflammation and Modulates Cognitive Dysfunction in Huntington’s Disease
Source: Biomolecules. 2021 Dec 27;12(1):34. doi: 10.3390/biom12010034 (PMC8773874; doi:10.3390/biom12010034)
Supplement: Supplementary file 1 [file biomolecules-12-00034-s001.zip › biomolecules-1492481-supplementary.pdf]

Supplementary material

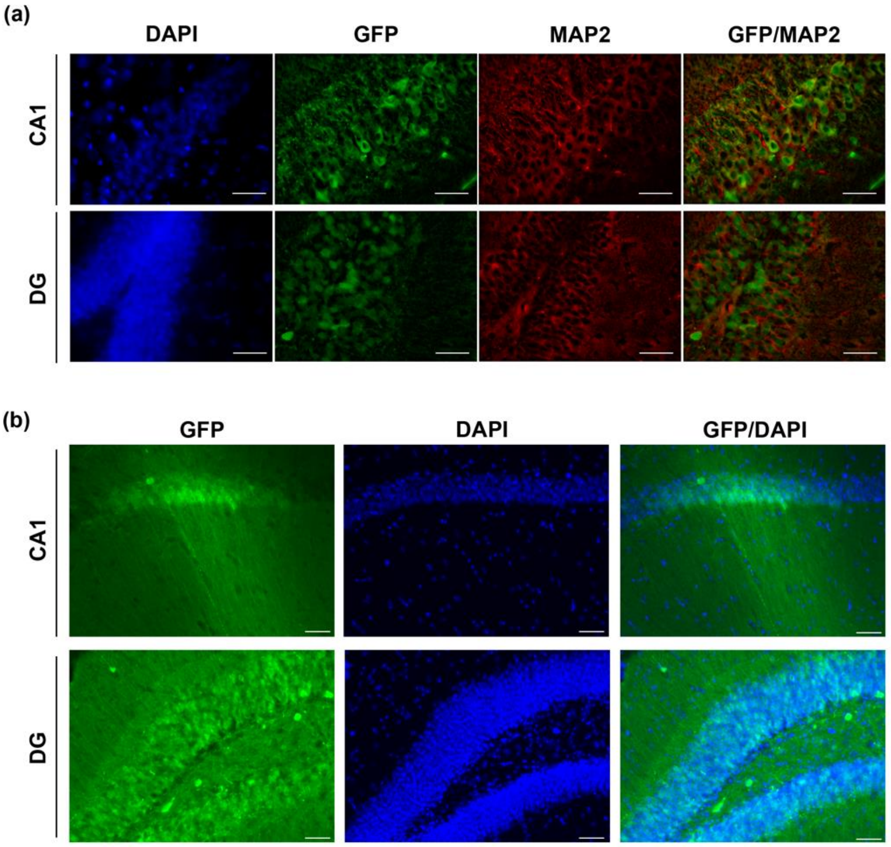

Figure S1. RTP801 hippocampal silencing is restricted to principal neurons Brain coronal sections were obtained after performing the behavioral battery (19-weeks old mice). Sections were stained against GFP protein (in green) and MAP2 (in red) (**a**) or GFP (green) (**b**) to show specific transduction of neuronal cells by the AAV serotype 2/8. Nuclei were stained with Hoechst33342 (in blue). Images were obtained from a WT shCt mouse dorsal hippocampus and were taken under epifluorescence microscopy. Scale bars, 20μm.

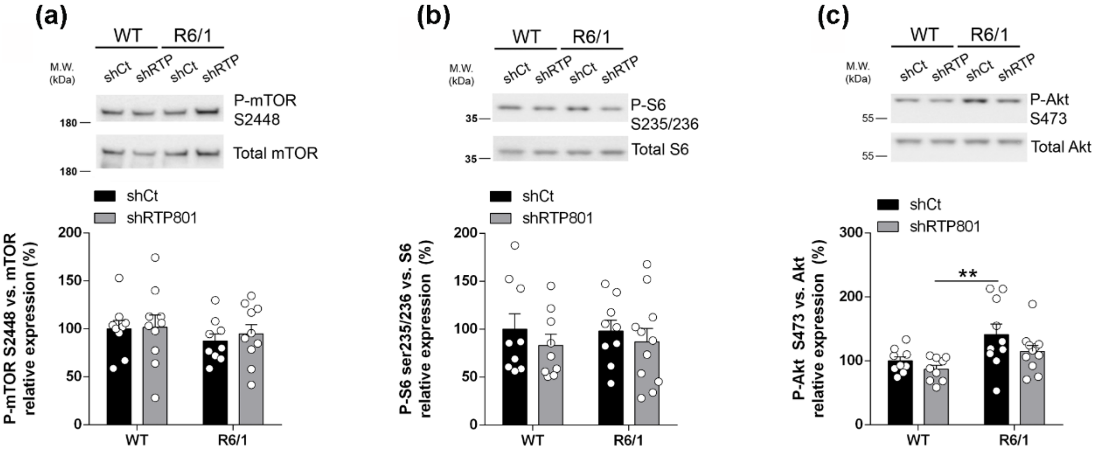

Figure S2. mTOR activity in dorsal hippocampal samples from WT and R6/1 mice injected with AAV. (a-d) Immunoblotting showing phosphorylated mTOR at Ser2448 (a), mTORC1 readout S6 at Ser235/236 (b) and mTORC2 readout Akt at Ser473 (c) and total proteins mTOR, S6 and Akt as loading controls. Graphs show the densitometric quantification of phosphorylated proteins vs. total levels expressed as the mean ± SEM (P-mTOR Treatment effect: $F_{(1, 34)}=0.2055$, $p=0.6532$; Genotype effect: $F_{(1, 34)}=0.9312$, $p=0.3414$; P-Akt Treatment effect: $F_{(1, 35)}=3.154$, $p=0.0844$; Genotype effect: $F_{(1, 35)}=9.757$, $p=0.0036$; P-S6 Treatment effect: $F_{(1, 34)}=1.081$, $p=0.3057$; Genotype effect: $F_{(1, 34)}=0.004331$, $p=0.9479$). All data were analyzed by two-way ANOVA followed by Bonferroni's post hoc test: ** $P<0.01$.

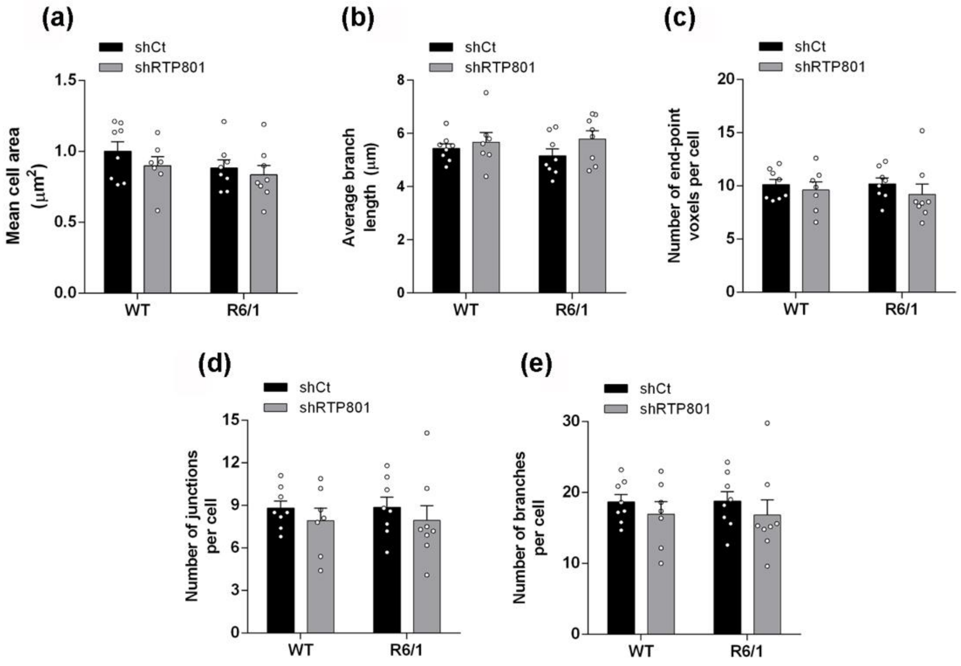

Figure S3. Microglial morphology in WT and R6/1 mice injected with AAV in the hippocampus. Quantitative analysis of microglial cell area (a), length of cell processes (b) and number of endpoints (c), junctions (d), and branches (e). Data are expressed as the mean ± SEM. All data were analysed by two-way ANOVA. No differences were observed in any group.

Table S1. Human postmortem HD brains. Neuropathological hallmarks are indicated: Vonsattel degrees, ranging from 1 to 4 and number of CAG repeats.

PATHOLOGICAL DIAGNOSIS	SEX	AGE (YEARS)	CAG REPEATS	HOURS POSTMORTEM
Normal	Male	83	-	13:00
Normal	Male	76	-	11:30
Normal	Male	58	-	5:00
Normal	Male	64	-	10:00
Normal	Male	86	-	10:15
Normal	Male	58	-	5:00
Normal	Male	86	-	7:25
Normal	Male	82	-	2:30
Normal	Female	83	-	7:30
Normal	Female	93	-	5:30
Normal	Female	83	-	7:33
Normal	Female	83	-	7:30
Normal	Female	97	-	7:20
Normal	Female	93	-	5:30
Normal	Female	83	-	7:20
HD, Vonsattel 3-4	Male	55	n.a.	7:00
HD, Vonsattel 3	Male	53	45 ± 2	7:00
HD, Vonsattel 3	Male	85	40	5:30
HD, Vonsattel 2	Male	76	20/41	6:00
HD, Vonsattel 2	Male	72	n.a.	13:10
HD, Vonsattel 2-3	Male	68	42±2	6:10
HD, Vonsattel 1	Male	73	40±2	7:00
HD, Vonsattel 3	Male	56	43	4:30
HD, Vonsattel 2-3	Male	84	39	8:00

HD, Vonsattel 2	Female	69	42	15:30
HD, Vonsattel 2	Female	86	40	12:20
